# Low Ankle-GO Score While Returning to Sport After Lateral Ankle Sprain Leads to a 9-fold Increased Risk of Recurrence: A Two-year Prospective Cohort Study

**DOI:** 10.1186/s40798-024-00693-w

**Published:** 2024-03-08

**Authors:** Brice Picot, François Fourchet, Ronny Lopes, Gauthier Rauline, Kinan Freiha, Pieter D’hooghe, Eugénie Valentin, Alexandre Hardy

**Affiliations:** 1 Interuniversity Laboratory of Human Movement Sciences, Savoie Mont-Blanc University, Chambéry, 7424, F-73000 EA France; 2 French Society of Sports Physical Therapist (SFMKS Lab), Pierrefitte-sur-Seine, France; 3 Physiotherapy Department, La Tour Hospital Swiss Olympic Medical Center, Meyrin, Switzerland; 4 Centre Orthopédique Santy, FIFA Medical Center of Excellence, Hôpital Privé Jean Mermoz, Groupe Ramsay, Lyon, France; 5 Clinique du Sport Paris, Paris, France; 6 Aspetar Orthopaedic and Sports Medicine Hospital, Doha, Qatar; 7 Département STAPS, Campus Technolac, Le Bourget-du-Lac, 73370 France

**Keywords:** Lateral ankle sprain, Return-to-sport, Reinjury, Risk factors, Ankle-GO

## Abstract

**Background:**

Lateral ankle sprain (LAS) is the most common sports injury, leading to a high rate of recurrence and the development of chronic ankle instability. One possible explanation is the lack of objective, evidence-based criteria to inform return to sport decisions following LAS. The aim of this study was therefore to assess the efficacy of a new functional score to distinguish patients at risk of recurrent LAS within two years after the initial injury.

**Methods:**

The Ankle-GO score was used in 64 active patients two months after LAS. This composite score includes 2 self-reported questionnaires and 4 functional tests, for a maximum score of 25 points. The rate of reinjury was prospectively recorded 2 years after inclusion. Potential predictive variables for reinjury were tested using the Chi-square and independent *t*-tests. The area under the receiver operating characteristics curve (AUC) with the optimal cut-off score was determined to assess the predictive value of the Ankle-GO score for the risk of reinjury. Multivariate logistic regression was then used to determine the influence of risk factors of reinjury.

**Results:**

Fifty-four (85%) patients were included (23 men and 31 women, 34.7 ± 13 years old) including 18 (33.3%) with a reinjury. The two-month Ankle-GO score was lower in patients with a recurrent LAS (5.4 ± 2.8 points vs. 9.1 ± 4.5, *p* = 0.002) and predicted the risk of reinjury (AUC = 0.75). Patients with < 8 points were found to have a significantly higher risk of reinjury (OR = 8.6; 95%CI: 2-37.2, *p* = 0.001). Women also tend to have a higher risk of recurrence (OR = 3.8; 95%CI: 0.9–15.5, *p* = 0.065).

**Conclusion:**

The Ankle-GO score is a new objective criterion for RTS after LAS. Patients with a low score at two months have a 9-fold greater risk of recurrence within two years.

## Key points

• The two-year risk of ankle sprain recurrence is nine times higher in patients with a low Ankle-GO score.

• Women tend to have a fourfold higher risk of re-injury within two years, despite an initial Ankle-GO score comparable to men.

• This innovative score could significantly influence the clinical management of LAS by providing an objective assessment of the readiness to RTS after LAS.

## Background

Lateral ankle sprain (LAS) is the most common sports injury. It is associated with a significant risk of recurrence, in particular a two-fold increased risk in the year following the initial injury [1, 2]. Moreover, about 40% of individuals develop chronic ankle instability (CAI) after their first LAS [3, 4]. One potential contributing factor to this high rate of recurrence and the consequences of LAS is a premature return to sport (RTS) [5]. Studies show that nearly 50% of patients RTS within three days after LAS, and 80% within a week [6, 7], despite lingering impairment and residual functional deficiencies [8].

There is no consensus on objective criteria for the RTS following LAS to date [2, 9] and this decision is usually time-based. A recent multidisciplinary consensus of international experts identified 5 domains as objective criteria for the evaluation of the RTS [5]. In addition to **P**ain severity and **A**nkle impairment, this consensus emphasized the importance of monitoring the **A**thlete’s perception, **S**ensorimotor control, and **S**port/functional performance. The Ankle-GO is a recently developed objective score [10] based on this “PAASS” framework and including various functional tests and patient self-reported questionnaires. These tests were chosen for their ability to differentiate CAI patients from copers and healthy individuals [11].

Although the Ankle-GO has been shown to reliably discriminate and predict RTS at the same level of play after LAS [10], its ability to identify patients at risk of recurrence has not yet been established. This study assessed whether the Ankle-GO score could predict the risk of reinjury following LAS. We hypothesized that the scores in patients with recurrent LAS within two years after injury would be lower at two-months post-LAS and predictive of the risk of re-injury. We also hypothesized that other established risk factors (age, sex and type or level of sports) [12] could influence the risk of reinjury.

## Methods

### Population

A priori power analysis showed that a minimum sample size of 54 participants (G*Power, Version 3.1, University of Dusseldorf, Germany) was needed to detect a moderate effect size for logistic regression (OR = 3.5), with a power of 0.80 and type 1 error of 0.05 [13, 14]. Considering a potential risk of 20% lost to follow-up patients [3], sixty-four patients (36 women and 28 men, 33.7 ± 13.2 years old) with recent LAS were included in the study (Table 1). The patients were all recruited in the same clinic, from January to August 2021. LAS was defined according to the International Ankle Consortium criteria as “an acute traumatic injury to the lateral ligament complex of the ankle joint from an excessive and sudden inversion mechanism of the rear foot or combined plantar flexion and adduction of the foot that prevents (the patient) from participating in sports” [15]. Only patients who practiced a sport at least once a week and who wished to RTS were included. The injury had occurred less than one month before inclusion, and patients were all initially examined by the same experienced orthopaedic surgeon. Exclusion criteria were the presence of a fracture and suspected syndesmosis injury (i.e. mechanism of injury involving dorsiflexion and external rotation of the foot, pain during palpation of the anteroinferior tibio-fibular ligament or the dorsiflexion lunge, a positive squeeze test) [16]. A prescription for personalized rehabilitation was given to the patients on the day of the consultation.

**Table 1:** Demographic characteristics

	Participants
Sex (men/women)	64 (36/28)
Age (years ± SD)	34.8 ± 13.2
**Type of sport, n (%)**	
Pivot contact	19 (29.7%)
Pivot	22 (34.4%)
In line	23 (35.9%)
**Level of practice, n (%)**	
Professional	2 (3.2%)
Intensive (> 6 h per week)	21 (32.8%)
Regular (2–6 h per week)	34 (53%)
Leisure (< 2 h per week)	7 (10.9%)

### Patient Follow-up

Two months after injury, all patients completed the Ankle-GO score supervised by the same experienced physical therapist who was not managing rehabilitation. The Ankle-GO is a valid, reliable, objective score [10] including 6 items selected on the basis of the PAASS framework from the International Ankle Consortium [5] and considered to be relevant for the monitoring of LAS patients [2, 9]. The total score is 25 points and the method of calculation is summarized in Table 2. Four tests in the Ankle-GO evaluate functional performance: the Single Leg Stance on a firm surface [17], the modified Star Excursion Balance Test [18, 19], the Side Hop Test [20], and the Figure-of-8 Test [21]. The two subscales of the Foot and Ankle Ability Measure to evaluate activities in daily life (FAAM_adl_) and sports (FAAM_sport_) [22], as well as the Ankle Ligament Reconstruction-Return to Sport after Injury (ALR-RSI) [23] were also used to assess the patient’s perception.

**Table 2:** Ankle-GO score calculation

	TESTS		RAW VALUES	POINTS	MAXIMUM SCORE
**FUNCTIONAL PERFORMANCE TESTING**	**Single leg stance test (SLS)**		> 3 errors	0	**3**
			1–3 errors	1	
			0 error	2	
			No apprehension	+ 1	
	**Star excursion balance test (SEBT)**		< 90%	0	**7**
			90–95%	2	
			> 95%	4	
			Anterior (ANT) > 60%	+ 1	
			Posteromedial (PM) > 90%	+ 1	
			No apprehension	+ 1	
	**Side hop Test (SHT)**		> 13 s	0	**5**
			10–13 s	2	
			< 10 s	4	
			No apprehension	+ 1	
	**Figure-of-8 hop Test (F8T)**		> 18 s	0	**3**
			13–18 s	1	
			< 13 s	2	
			No apprehension	+ 1	
**PATIENT REPORTED OUTCOME MEASURE**	**Foot and Ankle Ability Measure (FAAM)**	**Activities of Daily Living**	< 90%	0	**2**
			90–95%	1	
			> 95%	2	
		**Sport**	< 80%	0	**2**
			80–95%	1	
			> 95%	2	
	**Ankle ligament reconstruction-return to sport after injury (ALR-RSI)**		< 55%	0	**3**
			55–63%	1	
			63–76%	2	
			> 76%	3	
**Ankle-GO**	**25**				

**Fig. 1:**
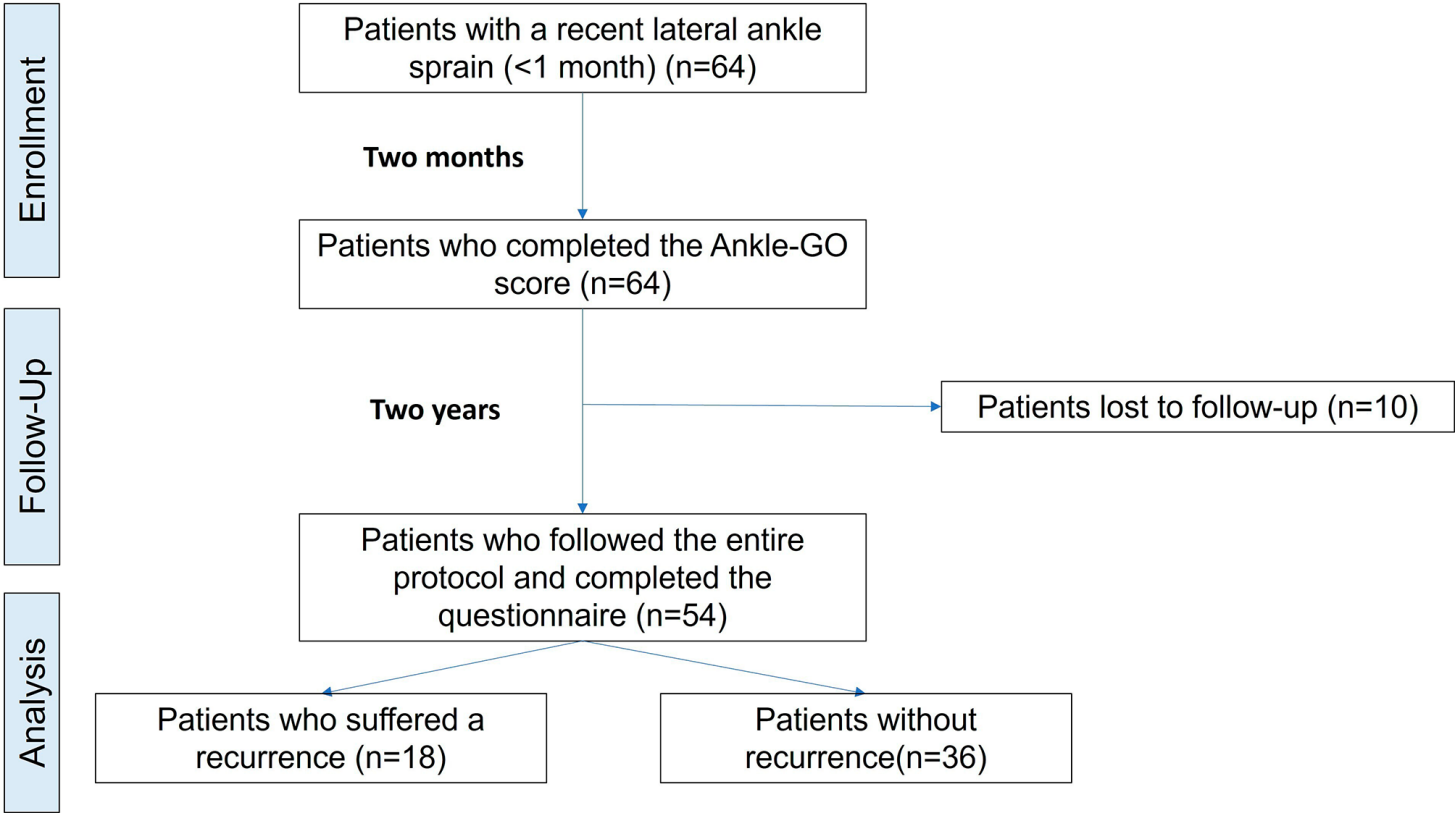
Flowchart of inclusion and analysis

Patients were contacted by a blind assessor two years after the initial LAS and asked if they suffered a recurrent LAS. Recurrent LAS was defined as a new ipsilateral LAS in the same location and of the same type [24]. The mechanism of injury was also reported (“contact” or “non-contact”). The study cohort is summarized in a flow chart. (Fig. 1). Patients provided informed consent, and this study received Institutional Ethics Approval (IRB00010835).

### Data Analysis

The analysis and presentation of data were consistent with the CHecklist for statistical Assessment of Medical Papers (CHAMP) [25]. There were two groups according to the recurrence or not of LAS (primary outcome). Data were checked for normality and homogeneity of variance. The relationship between potential predictive variables and recurrent LAS was tested using the Chi-square test for categorical measurements and the Mann-Whitney or independent *t*-tests. Variables with a P value < 0.20 were considered for further analysis in logistic regression. The discriminant validity of all items and the total Ankle-GO score was assessed using independent t-tests and Cohen’s d effect sizes with 95% confidence intervals (CIs) between patients with recurrent LAS at two years and those without. Effect sizes were interpreted as small: d = 0.20–0.49, moderate: d = 0.50–0.79, and large: d ≥ 0.80 [26].

The predictive validity of the two-month Ankle-GO score to identify patients who would have a recurrent LAS was also evaluated with the receiver operating characteristic curve. The area under the curve (AUC) was determined with a precision score considered to be null (AUC = 0.5), low (0.5 < AUC < 0.7), fair to good (0.7 ≤ AUC < 0.9), high (0.9 ≤ AUC < 1), or perfect (AUC = 1). The optimal cut-off score was calculated using the Youden index (J = sensitivity + specificity − 1). Variables were then recoded into dichotomous variables that were either above or below the cut-off point to simplify interpretation of risk factors and the related odds ratios [27, 28]. Because other factors could influence the risk of reinjury [12], multivariate logistic regression (stepwise method) was performed to determine whether the chosen variables (i.e. only those with a predictive p value < 0.20) were associated with reinjury [28, 29]. Odds ratios (OR) and 95% CI) were reported for the variables associated with an increased risk of reinjury. The statistical analysis was performed using JASP (Amsterdam 0.12.2.0). The level of significance was set at 0.05.

## Results

Two years after the initial LAS, fifty-four patients (85%) responded to the survey. Eighteen (33.3%) of these suffered a recurrent LAS (Table 3). All of these were non-contact injuries. To determine the potential ability of the parameters to LAS recurrence, we first identified the variables that showed differences between the two groups (injured vs. non-injured). In total, 9 variables met the criterion of *p* < 0.20: the two FAAM subscales, all of the SEBT components (ANT, PM, PL and COMP), the SHT, sex and the Ankle-GO score (Tables 3 and 4).

**Table 3:** Comparison between injured and non-injured patients at the end of the follow up period

Lost to follow-up	10/64 (15%)		
Reinjury (*n* = 54)	YES, *n* = 18 (33.3%)	NO, *n* = 36 (66.6%)	*p* value
Sex (women/men)	18 (4/14)	36 (19/17)	**0.032**
Age (years ± SD)	36.8 ± 9.7	34.3 ± 14.7	0.51
Ankle-GO (points ± SD)	5.4 ± 2.8	9.1 ± 4.5	**0.002**
Type of reinjury	18 ankle sprains (100% non-contact)		
**Type of sport, n (%)**			
Pivot contact	7 (38.9%)	8 (22.2%)	0.259
Pivot	2 (11.1%)	10 (27.8%)	
In line	9 (50%)	18 (50%)	
**Level of sport, n (%)**			
Professional	1 (5.6%)	1 (2.8%)	0.794
Intensive (> 6 h per week)	7 (38.9)	11 (30.5%)	
Regular (2–6 h per week)	8 (44.4%)	21 (58.6%)	
Leisure (< 2 h per week)	2 (11.1%)	3 (8.3%)	

**Table 4:** Mean (± SD) Ankle-GO scores at two months in patients with a recurrent LAS two years after the initial LAS and in those without

	Injured	Uninjured	p-value	Cohen’s d	95% CI Lower limit	95% CI Upper limit
FAAM_adl_ (%)	78.6 ± 14.4	87.2 ± 14.8	**0.046**	0.3	0.01	1.17
FAAM_sport_ (%)	51.4 ± 17.2	61.3 ± 23.9	0.124	0.29	-0.12	1.02
ALR-RSI (%)	40.6 ± 21	46.1 ± 22.3	0.386	0.29	-0.32	0.82
SLS (errors)	3.7 ± 3.2	3.1 ± 2.7	0.447	0.29	-0.79	0.35
SEBT Comp (%)	76.6 ± 8.2	81.2 ± 6.8	**0.035**	0.3	0.05	1.20
SEBT Ant (%)	57.7 ± 7.2	61.7 ± 6.2	**0.039**	0.3	0.03	1.19
SEBT PM (%)	88.9 ± 9.6	93.7 ± 7.9	0.053	0.3	-0.01	1.12
SEBT PL (%)	83 ± 11.8	88.8 ± 9.9	0.065	0.3	-0.03	1.12
SHT (s)	25 ± 8.2	20.1 ± 12.5	0.138	0.29	-1.01	0.14
F8T (s)	23.3 ± 9.6	19.9 ± 9.7	0.234	0.29	-0.92	0.22

FAAM_adl−sport_= Foot and Ankle Ability Measures-Activities of daily living & sport subscales; ALR-RSI = Ankle Ligament Reconstruction Return to Sport after Injury; SLS = Single Leg Stance; SEBT = Star Excursion Balance Test; Comp = Composite score, Ant = Anterior, PM = posteromedial, PL = posterolateral; SHT = Side Hop Test; F8T = Figure of eight test

**Fig. 2:**
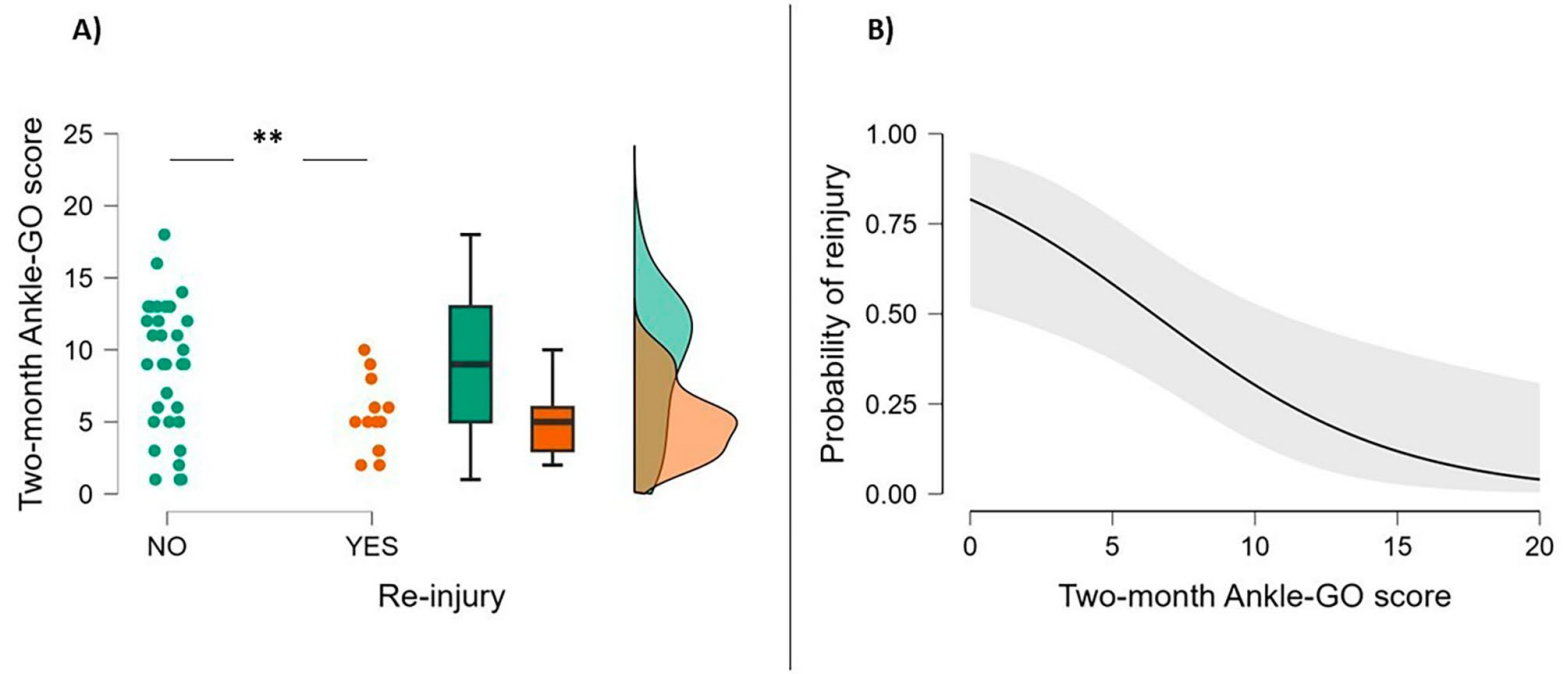
**(a)** Ankle-GO scores at two months in patients with a recurrent LAS within two years and those without **(b)** Estimate plot of the probability of recurrence. **p < 0.01

The Ankle-GO score at two months was significantly lower in patients with recurrent LAS (*p* < 0.002) (Fig. 2; Table 3). Of all the items of the Ankle-GO score, only the FAAM_adl_, anterior direction and the SEBT composite score were found to be significantly lower in patients with recurrent LAS (Table 4).

The predictive capacity of the 2-month Ankle-GO score to identify patients who would have a recurrent LAS within 2 years after the initial injury was good (AUC = 0.75; 95% CI: 0.62–0.88; *p* = 0.003). A Youden index of 0.47 was observed for a cut-off score of 8 points, corresponding to a sensitivity of 83% and a specificity of 64%. Thus, Ankle-GO scores were re-coded as being either above or below this cut-off point. The full model containing all predictors (Ankle-GO score and sex) was statistically significant (*p* < 0.001), indicating that the model identified participants at a higher risk of having a recurrent LAS during follow-up. The model explained between 25% (Cox and Snell R²) and 34% (Nagelkerke R²) of the variance in recurrent LAS’s and correctly classified 78% of cases.

The Ankle-GO score was the only statistically significant contribution to the model on multivariate logistic regression, with an OR of 8.6 (95% CI: 2 to 37.2, *p* = 0.004). That is, patients who scored below 8 points on the Ankle-GO score two months after an initial LAS had a nearly 9 times greater risk of reinjury within the next two years. There was also a trend (OR = 3.8; 95% CI: 0.9 to 15.5, *p* = 0.065) showing that woman had a risk of reinjury that was almost 4 times greater than men, controlling for other factors in the model.

## Discussion

The main objective of this study was to evaluate the capacity of the Ankle-GO score to predict the risk of recurrent LAS two years after the initial injury. First, our results confirmed a high rate of recurrence, with up to 30% of patients reporting a new LAS [15, 30, 31]. The cause of this high rate of reinjury is often explained by poor management of RTS [5] as no evidence-based criteria have been published to date to help make this decision [2, 9]. The goal of the Ankle-GO score is to address this need, and assist clinicians in the decision-making process [10].

The present study showed that the two-month Ankle-GO score effectively predicts the two-year post-LAS risk of recurrence. Scores were significantly lower in patients with reinjury than in those without (Table 3), with a difference between the two groups that was greater than the established minimum detectable change (1.2 points) [10]. In particular, patients with a score < 8 points on the Ankle-Go scale had a nearly 9 times greater risk of recurrence during the first two years after LAS.

To the best of our knowledge, this is the first objective RTS criterion to identify individuals with an increased risk of reinjury following LAS. Interestingly, the same cut-off score (8-points) was reported to identify patients who will return to their preinjury level of sports 4 months after LAS, with a sensitivity of 67% and a specificity of 92% [10]. This ability to predict both the level of play after the RTS and the risk of reinjury is due to the multidimensional design of the Ankle-GO. Because the causes of reinjury and the development of CAI are multifactorial [12], this score was designed to provide a comprehensive assessment, including various components to evaluate all potential deficits associated with LAS and CAI [4, 5, 32]. The selection of items and the rating system for the Ankle-GO score (Table 2) was described in a previous study [10]. In summary, the score includes several items (questionnaires and functional tests) that were chosen according to the PAASS framework [5], clinical guidelines [33, 34] and systematic reviews with expert opinions on RTS [2, 9]. All items were selected based on their relevance, reliability and ability to differentiate CAI patients from copers and healthy individuals [11]. The Ankle-GO is a valid and reliable score [10] including 2 patient-reported outcome measures (FAAM and ALR-RSI), assessing perceived ankle confidence and psychological readiness to RTS [23, 35]. Low scores on these questionnaires are strongly associated with a poor prognosis and greater disability in those with CAI [36, 37]. Functional assessments are highly recommended for the management of LAS patients [4, 33]. Thus, the Ankle-GO score also includes 4 functional tests to evaluate postural control, hopping, jumping and agility, which are frequently impaired following LAS and CAI [2, 5, 9, 38]. Furthermore, and as recommended by Caffrey et al., [21] the feeling of instability reported during the tests was also considered when calculating the Ankle-GO.

When comparing the two-month scores, only the FAAM_adl_ and SEBT scores were significantly lower in patients with a recurrent LAS (Table 4). Because no single component of the Ankle-GO score independently predicted the risk of reinjury, the use of combined scores rather than single evaluations are advisable in patients with LAS. It is important to note that the two-month values of all of the Ankle-GO items were below the criteria used to define CAI (FAAM_adl_ scale, ˂90% and FAAM_sport_ scale, ˂80%) [15] in both groups. Similarly, the mean functional test values identified poor balance control during the SLS (> 3 errors) [39] and SEBT (composite score < 89.6%) [40], as well as slow agility test performances (> 12.8s on the SHT and > 17.36s on the F8T) [11, 39] in all patients, whatever their reinjury status. Only posteromedial performance on the SEBT exceeded the cut-off score of (> 91%) [39] in patients with no recurrent LAS, although the difference with the injured group was not significant (93.7 ± 7.9 vs. 88.9 ± 9.6 respectively, *p* = 0.053). This confirms the study by Mcann et al., [8] which showed that the resolution of structural and functional impairment was incomplete, and revealed LAS-related activity limitations at the time of RTS (after an average of 12 days). Although the RTS occurred within a week following LAS in 80% of patients [6, 7], this decision should be based on objective criteria rather than time-related considerations.

Very few prospective studies have evaluated the role of sex in the risk of recurrence in patients with an initial LAS [41]. Results show the risk of reinjury is almost 4 times higher in women than in men, with no baseline differences in the two-month Ankle-GO score (7.7 vs. 8 points, *p* = 0.8, respectively). Despite conflicting evidence on the influence of sex on LAS [12], women seem to be at a higher risk of LAS [33, 42, 43] and of developing CAI [44] than men. A recent study has also shown that there was a higher risk of recurrence in women following ankle surgery [45]. Because of these differences, clinicians should use the Ankle-GO score for both sexes but be more cautious about low scores in women. Unlike previous results, age was not identified as a risk factor for reinjury in our study [12, 46]. Several studies have shown that patients under the age of 24 were at a higher risk of LAS [28, 47]. In the current study, 13 patients (20%) were under 25, and only one had recurrent LAS. Further larger studies targeting adolescents should be performed using the Ankle-GO score to reduce the risk of reinjury.

The socioeconomic costs of poor management of initial LAS can be high because of the high rates of recurrence and CAI [30]. The economic burden of suboptimal care and follow-up after initial LAS has numerous causes including the costs of direct and indirect healthcare and long-term rehabilitation as well as the loss of productivity and quality of life from CAI which diminishes the individual’s ability to perform physical activities and potentially leads to a sedentary lifestyles with associated health problems [48, 49]. A long-term analysis of costs after LAS is essential to assess treatment efficacy, and to determine if it is cost-effective and associated with a favourable clinical outcome [30].

The use of the free application Ankle-GO (https://anklego.com/) during the late phase of rehabilitation allows to quickly identify, in a clinician-friendly and reliable way, patients who are at risk of a new injury.

### Strength and Limitations

To our knowledge, this is the first prospective study to evaluate the value of objective RTS criteria to predict the risk of reinjury within two years. However, this study has certain limitations. First, it is well known that the risk of reinjury after LAS is multifactorial [12]. Although age, sport and level of play were included in the predictive model and did not influence recurrence, we did not consider all known risk factors of LAS reinjury. For instance, although the severity of the injury was systematically assessed by the surgeon (pain, swelling, ligament laxity) this was not considered in the analysis. Indeed, very recently Netterstrom et al. 2022 in their meta-analysis reported limited and contradictory evidence that clinical tests can provide an accurate assessment of injury severity [50]. Previously, Pourkazemi et al. in their systematic review raised concerns about the validity of grading systems based solely on symptoms [51]. Furthermore, they revealed that the severity of the initial ankle sprain does not necessarily predict re-injury.

The exact number and content of rehabilitation sessions for each patient was not controlled and may have influenced the results. As the International Ankle Consortium guidelines recommend physical therapy on a case-by-case basis according to each patient’s specific individual deficits [32], a detailed analysis of the type of rehabilitation was beyond the scope of this study. It would therefore seem appropriate to assess the influence of rehabilitation content on the Ankle-Go score at the time of RTS.

In addition, reinjury during follow-up was based on a single survey at two years. Thus, the number, severity and exact timing of recurrence were not examined. This study is an important step in establishing objective RTS criteria after LAS for secondary prevention. However, it is important to note that the Ankle-GO does not include all the items of the PAASS framework [5]. It could be important to combine this score with other measures such as strength, range of motion or neurocognitive assessments [52] in order to obtain a broader overview of patients’ deficits. Further studies using the Ankle-GO score are needed to assess its ability to identify potential copers (secondary prevention), predict the development of CAI (tertiary prevention), as well as the occurrence of a first ankle sprain in a healthy population (primary prevention) [53, 54].

### Clinical Implications

The decision to RTS should not be primarily based on time but on objective criteria. The Ankle-GO score is a cost-effective, rapid (takes less than 20 min), and user-friendly tool for clinicians.

The Ankle-GO score includes various components that target critical outcomes associated with LAS to allow clinicians to identify remaining impairment and reduce the risk of reinjury. During the later stages of rehabilitation and on the RTS continuum, it can provide a goal-oriented assessment similar to the clinical assessment of acute lateral ankle sprain injuries [32]. For example, dynamic postural control exercises should be prescribed in patients with a low Ankle-GO score and reduced SEBT values to manage remaining deficits.

## Conclusions

A low Ankle-GO score is associated with an increased risk of reinjury within 2 years after LAS. Patients with a score < 8 points two months after the initial LAS had a 9 times greater risk of reinjury. In addition, there was a trend towards a higher risk of re-injury in women. The Ankle-GO score is the first objective tool to help clinicians objectively evaluate patients for the RTS. Further studies are needed to assess the predictive value of the Ankle-GO score for the development of CAI.

## Data Availability

Data are available upon reasonable request. Please contact: alexandre.hardy@me.com.

## Abbreviations

LAS: Lateral Ankle Sprain
RTS: Return to Sport
CAI: Chronic Ankle Instabily
ALR-RSI: Ankle Ligament Reconstruction-Return to Sport after Injury
PAASS: Pain, Ankle impairments, Athlete perception, Sensorimotor control, Sport performance
CHAMP: CHecklist for statistical Assessment of Medical Papers
OR: Odds ratios
95% CI: 95% confidence intervals
AUC: Area Under the Curve
FAAM_adl−sport_: Foot and Ankle Ability Measures-Activities of daily living & sport subscales
SLS: Single Leg Stance
SEBT: Star Excursion Balance Test
Comp: Composite score
Ant: Anterior
PM: Posteromedial
PL: Posterolateral
SHT: Side Hop Test
F8T: Figure of eight test
